# Two codes of RNA editing by deamination in human diseases

**DOI:** 10.1038/s12276-025-01633-8

**Published:** 2026-02-18

**Authors:** Dong Jun Min, Suyeon Lee, Young-suk Lee, Jun Cho

**Affiliations:** 1 Department of Biomedical Science and Engineering, Gwangju Institute of Science and Technology, Gwangju, Republic of Korea; 2 Department of Bio and Brain Engineering, Korea Advanced Institute of Science and Technology, Daejeon, Republic of Korea; 3 Graduate School of Engineering Biology, Korea Advanced Institute of Science and Technology, Daejeon, Republic of Korea

**Keywords:** Molecular biology, Immunology, Neuroscience, Biochemistry, Cancer

## Abstract

RNA editing is a post-transcriptional modification that expands transcriptomic and proteomic diversity. Advances in high-throughput sequencing across a broad range of biological and pathological contexts have enabled systematic identification of editing events driven by two major RNA deaminase families: ADAR and APOBEC, which catalyze adenosine-to-inosine (A-to-I) and cytidine-to-uridine (C-to-U) substitution, respectively. Genome-wide profiling of RNA editing has uncovered a substantial number of differentially edited loci in various conditions, implicating the post-transcriptional events in physiological and pathological regulation. Aberrant RNA editing alters the functional information of coding and noncoding transcripts, perturbing protein activity, RNA stability and other gene expression programs, which contributes to immune imbalance, viral infection, neurological impairment, metabolic disorders and tumorigenesis. The two codes of A-to-I and C-to-U RNA editing harbor common potential for single base conversion with varied expression of responsible enzymes across many physiological and pathological conditions. Here we provide a comprehensive and parallel overview on ADAR-mediated A-to-I and APOBEC-mediated C-to-U editing, with emphasis on their molecular mechanisms, physiological roles and pathological dysregulation in human health and disease.

## RNA editing reveals insights into health and disease

RNA editing is a process that changes the genetic information in RNA molecules after they are made from DNA. The study explores two main types of RNA editing: adenosine-to-inosine (A-to-I) and cytidine-to-uridine (C-to-U). Researchers aim to understand how these processes affect human health and disease. The study reviews how RNA editing works and its role in various biological functions. It uses data from human and mouse genetics to explore how RNA editing impacts immunity, viral infections, brain function, metabolism and cancer. The researchers highlight that A-to-I editing is common in many tissues, while C-to-U editing is more specific to certain tissues. The findings show that RNA editing can influence disease development, such as cancer progression and immune system disorders. The researchers conclude that understanding RNA editing could lead to new treatments for diseases.

This summary was initially drafted using artificial intelligence, then revised and fact-checked by the author.

## Introduction

RNA editing is a type of RNA modification that alters a single nucleotide of the RNA molecule after it has been transcribed from DNA. This single-nucleotide alteration recodes the genetic information and leads to the translation of non-encoded protein variants. RNA editing was first discovered in 1986 through sequencing of the mitochondrial DNA and RNA of a parasitic kinetoplastid named *Trypanosoma brucei*, the causative agent of sleeping sickness^1^. They found four uridine nucleotides in the RNA molecule that were not encoded in its corresponding DNA sequence. Importantly, this post-transcriptional insertion is critical as it corrects a codon frameshift for the production of subunit II of cytochrome oxidase (coxII). In human, RNA editing is commonly referred to as the site conversion of adenosine-to-inosine (A-to-I) mediated by ADAR1^2–4^. Inosine is known to be recognized as guanosine, which then results in the recoding of genetic information. The importance of A-to-I editing has been reported in multiple physiological contexts such as immunity^5,6^, viral infection^7^, neurobiology^8,9^ and metabolism^10^. Advances in sequencing technology have led to the transcriptome-wide discovery of A-to-I editing sites^11,12^ and enabled the exploration of A-to-I editing activity in human diseases^13^ and cancer biology^3,14^.

The other, less-known code of RNA editing in human is the site conversion of cytidine to uridine (C-to-U). C-to-U RNA editing was first discovered in 1987 when trying to understand the molecular mechanism of how the gene for apolipoprotein B (APOB) is able to produce a full-length isoform APOB-100 in the liver but a shorter, truncated isoform APOB-48 in the small intestine^15^. Of note, the imbalance between APOB-100 and APOB-48 production has been implicated in genetic disorders such as familial hypobetalipoproteinemia^16^. They found that a single cytidine in the *APOB* mRNA is converted to a uridine that creates a premature stop codon (UAA), resulting in the translation of the shorter, truncated isoform. The responsible enzyme for C-to-U RNA editing was later discovered in 1993 and named APOBEC1, which stands for apolipoprotein B mRNA editing enzyme catalytic subunit 1^17,18^. However, this second code of C-to-U RNA editing is less known, probably because the APOBEC1 and other APOBEC family proteins are also responsible for the site conversion of DNA molecules^19^. APOBEC-mediated DNA editing is key in the innate and adaptive immune systems including antiviral hypermutations in the HIV genome, restriction of retrotransposon activity and somatic hypermutation in immunoglobulin genes for the creation of diverse antibodies^19^. APOBEC-mediated DNA mutations represent a major axis of cancer biology, with misregulation of APOBEC family proteins thought to be responsible for the mutational signatures observed in most human cancer types^19–21^. This dual enzymatic role of APOBEC family proteins has thus challenged the community in distinguishing its role in RNA editing and DNA mutations in human diseases.

In this Review, we attempt to unravel the complexity of RNA editing in human diseases by (1) delineating its exact molecular mechanism and its function beyond recoding, (2) describing its role in physiology and pathology based on mouse and human genetics, and (3) structuring its large-scale dysregulation in cancer biology with a focus on breast cancer, glioblastoma (GBM) and leukemia. In particular, we deliberately organized this Review to present both A-to-I and C-to-U RNA editing in parallel, providing a holistic perspective on the two types of RNA editing in human diseases.

## Molecular mechanism of RNA editing

RNA editing is a natural mode of post-transcriptional regulation that modifies the sequence identity of endogenous RNA transcripts. The two most common types of RNA editing are A-to-I editing by ADAR family proteins and C-to-U editing by APOBEC family proteins. The molecular mechanism and target specificity of these two natural modes of RNA editing have been studied and reviewed largely in an independent manner^2,19^. In this section, we compare insights from both ADAR and APOBEC proteins to provide a complementary and comprehensive molecular perspective on RNA editing. We start by highlighting that the enzymatic mode of both RNA editing proteins is the removal of an amino group from RNA bases. This removal leads to alterations in the genetic information (also known as recoding) and intra- and intermolecular interactions of the RNA target. The biochemistry and target specificity of these two enzymatic modes are distinct at the molecular level, underlying the large functional diversity and versatility of RNA editing.

### ADAR family proteins and A-to-I editing

**Fig. 1:**
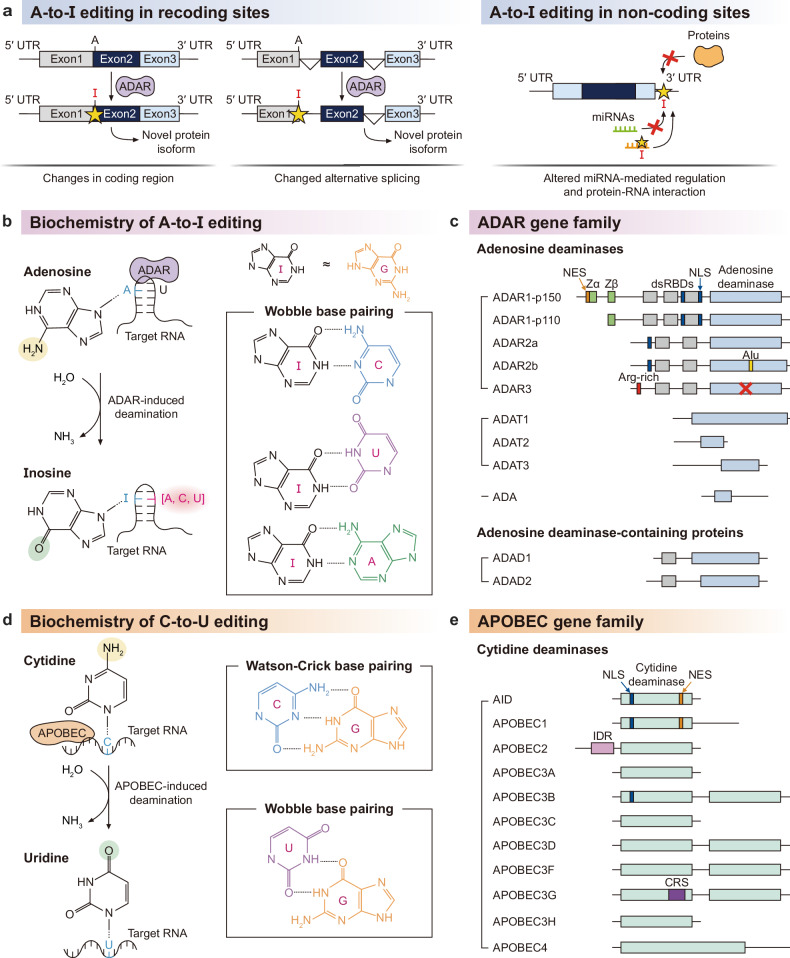
Molecular mechanisms and consequences of RNA deamination. **a** The functional impact of A-to-I RNA editing depends on its transcriptomic location. In coding regions, inosine can generate novel protein variants by altering the encoded amino acid sequence or splicing regulatory code. In noncoding regions, it may modulate protein–RNA interactions, and miRNA-mediated regulation. **b** Inosine is primarily interpreted as guanosine by splicing and translational machinery. It also permits promiscuous wobble base pairing which leads to a remodeling of the RNA–RNA interaction network. **c** A-to-I editing is catalyzed by the ADAR family which contains a catalytic adenosine deaminase domain. Other related protein families include ADA, ADAD and ADAT. **d** The other major form of RNA editing is the deamination of cytidine to uridine. A U–G wobble base pair is thermodynamically comparable to a canonical C–G pair, and so this C-to-U conversion is thought to have a minimal structural impact. **e** In mammals, C-to-U editing is catalyzed by the APOBEC gene family. The 11 human APOBEC members each display unique biological functions via distinct protein domains. NLS, nuclear localization signal; IDR, intrinsically disordered region; CRS, cytoplasmic retention signal.

The site-specific conversion of adenosine to inosine (A-to-I) is considered to be one of the most prevalent forms of RNA editing in *Caenorhabditis elegans*^22^, *Drosophila*^23^, zebrafish^24^, *Xenopus*^25^, mice^26,27^ and humans^2^^,12^^,28^. In human, it has been initially reported that 12,723 sites of 1,637 RNA transcripts undergo A-to-I editing^2^. Analyses of RNA sequencing data from the Genotype-Tissue Expression (GTEx) project suggest that the profiles of A-to-I editing are somewhat correlated across different tissues, with the exception of skeletal muscle and multiple brain regions^29^. Large-scale databases such as DARNED^30^, RADAR^31^ and REDIportal^12^ provide a catalog of 16 million putative A-to-I editing sites in human. These conversion sites are commonly categorized by functional impact (for example, recoding or noncoding) depending on whether they are located in the coding region or not (Fig. 1a). Inosine sites in the coding region are thought to be interpreted as guanosine and thus may lead to nonsynonymous mutations and the generation of recoded protein variants^11,32^. Recent improvements in the computational analysis revealed 1,517 editing sites within the coding region and their tissue specificity and site conservation^11^. Noncoding A-to-I editing is predominant in Alu-containing RNAs^2^ and unwinds double-stranded RNA (dsRNA) structure^33,34^. Other noncoding sites have been reported to alter tissue-specific microRNA (miRNA)-mediated regulation^35,36^, protein–RNA interaction^37^, and alternative splicing^38,37^.

From a molecular perspective, a site conversion to inosine is a removal of the amino group of adenosine that leads to a complete rewiring and expansion of inter- and intramolecular interactions of the RNA target^39^. The chemical structure of inosine is quite different from adenosine and instead resembles a guanosine without its 2-amino group (Fig. 1b). Optimal melting studies report that internal I–U base pairs are 2.3 kcal/mol less stable than A–U base pairs^40^, but I–C base pairs are 4.1 kcal/mol more stable than A–C base pairs^41^. Beyond Watson–Crick base pairs, ribosome crystal structures revealed that inosine can form wobble base pairs with adenosine and uridine^42^. The stochasticity of inosine intermolecular interactions has been reported in the context of reverse transcription^43^. In terms of mRNA translation, mass spectrometry studies have demonstrated that inosine can be interpreted as adenosine and even uridine^44^. In fact, the inosine codon IAC has been reported to be alternatively decoded as AAC at a rate of 25% under in vitro translation conditions.

A-to-I RNA editing is mainly catalyzed by enzymes encoded by the adenosine deaminase acting on RNA (*ADAR*) gene family (Fig. 1c). In 1987, Brenda L. Bass and Harold Weintraub discovered an enzyme in *Xenopus laevis* that could unwind dsRNA, which was later revealed to be a result of A-to-I RNA editing^33,45,46^. The *ADAR* gene family is thought to be evolved from the adenosine/AMP deaminase (*ADA*) superfamily^47^, which also includes the adenosine deaminase acting on the tRNA (*ADAT*) gene family^48,49^ and the adenosine deaminase domain-containing (*ADAD*) gene family^50^. ADAT1 (HGNC:228) is responsible for tRNA deamination at position 37 and involved in tRNA stability and structure^48^. ADAT2 (also known as dJ20N2.1, TAD2; HGNC:21172) and ADAT3 (also known as TAD3; HGNC:25151) form a heterodimer and edits the wobble position in the tRNA anticodon loop^51^. ADAD1 (also known as Tenr; HGNC:30713) and ADAD2 (also known as TENRL, FLJ00337; HGNC:30714) are highly tissue specific and RNA binding, but they lack the essential catalytic residues in their deaminase domains^52^. In this Review, we will focus on ADAR-mediated A-to-I editing.

In mammals, there are three members of the *ADAR* gene family: adenosine deaminase RNA specific (ADAR, also known as ADAR1, DRADA; HGNC:225), adenosine deaminase RNA specific B1 (ADARB1, also known as ADAR2, DRADA2, RED1; HGNC:226) and adenosine deaminase RNA specific B2 (ADARB2, also known as ADAR3, RED2; HGNC:227). ADAR1 is ubiquitously expressed and contains three tandem dsRNA-binding domains (dsRBDs), each with distinct target specificity: dsRBD1 is thought to be involved in substrate recognition and homo-/heterodimer complex formation^53^, dsRBD2 recognizes 16 base pairs of dsRNA with no apparent sequence specificity^54^, and dsRBD3 is responsible for stable homodimer formation and modulates editing efficiency^55^. Unlike ADAR1, ADAR2 and ADAR3 are tissue specific and contain two tandem dsRBDs that are responsible for the recognition of short and specific dsRNA structures such as the GRIA2 Q/R site and CYFIP2 K/E recoding^56–58^. The respective dsRBD1s of ADAR2 and ADAR3 share 73.13% sequence similarity. ADAR3 contains multiple mutations in its catalytic glutamate residue and is thought to be a competitive inhibitor of ADAR2^59^^,^^60^. All three ADAR genes encode a nuclear localization signal (NLS) that is responsible for their nuclear subcellular localization^61^.

There are two major isoforms of ADAR1 produced via alternative transcription start sites^62^. ADAR1-p110 is a 110-kDa isoform that is constitutively expressed in most cell types and is thought to play a role in nuclear RNA surveillance^63^. The shared Zβ domain binds to the Z-RNA (left-handed helical conformation of dsRNA) and thus provides another layer of substrate recognition^64,65^. Unlike the short isoform, the 150-kDa long isoform ADAR1-p150 is interferon-inducible and contains a nuclear export signal (NES) as well as an additional Z-RNA binding domain (Zα)^66,67^. The Zα is thought to be responsible for the A-to-Z conformational transition and the molecular basis for A-to-I RNA editing of inverted Alu repeats in the cytoplasm^68^.

### AID/APOBEC family proteins and C-to-U editing

The other major code of RNA editing is C-to-U: the removal of an amino group from cytidine that results in a nucleoside conversion to uridine. C-to-U RNA editing sites are found in both plants and mammals but regulated through completely different molecular mechanisms^69^. Plant C-to-U editing occurs in the mitochondria and chloroplasts and is reviewed in detail elsewhere^69^. As with A-to-I editing, there have been multiple attempts to discover and map mammalian C-to-U RNA editing sites from high-throughput RNA sequencing data^70,71^. Unlike A-to-I editing, C-to-U RNA editing is thought to be highly tissue specific such as in the gastrointestinal tract and multiple immune lineages^72,73^. However, rigorous data analysis of C-to-U RNA editing raises the possibility of a large number of false positives due to inherent limitations of the sequencing technology^71,74^. Beyond the coding region, C-to-U RNA editing sites are also enriched in the 3′ untranslated region (UTR), adding another layer of post-transcriptional regulation^70,71^.

Similar to A-to-I editing, the C-to-U site-specific conversion leads to recoding of genetic information and potential rearrangement of inter- and intramolecular interactions. However, this molecular rearrangement is considered subtle rather than dramatic, as both cytidine and uridine are pyrimidine nucleosides (Fig. 1d). U–A base pairs are more stable than C–A base pairs^75^. Nonetheless, the thermodynamic stability of U–G wobble base pairs is comparable to standard C–G Watson–Crick base pairs^76^, suggesting that C-to-U editing may not result in large disruption of RNA structure, miRNA-mediated interactions or any other noncoding regulations. That being said, the arrangement of hydrogen bonds of U–G wobble base pairs forms unique grooves with specific ligand-binding properties and slight distortion of the RNA helix^77^. In fact, U–G wobble base pairs were originally found via X-ray analysis of yeast tRNA structures^78–80^.

Mammalian C-to-U RNA editing is catalyzed by enzymes belonging to the apolipoprotein B mRNA editing catalytic polypeptide-like (*AID*/*APOBEC*) gene family^70,74^ (Fig. 1e). ApoB mRNA editing enzyme catalytic subunit 1 (APOBEC1, also known as BEDP, CDAR1, HEPR; HGNC:604) is responsible for the C-to-U editing of *APOB* mRNAs that leads to the codon conversion of Gln (CAA) to a stop codon (UAA)^81^. The catalytic domain of *AID*/*APOBEC* family proteins is considered to be distantly related to highly conserved cytidine deaminase (CDA also known as CDD, HGNC:1712) which acts on free pyrimidine bases or nucleosides as part of the pyrimidine salvage pathway^82,83^. Not all members of the *AID*/*APOBEC* gene family undergo RNA deamination^19,73,84^. Activation-induced cytidine deaminase (AICDA, also known as AID, HIGM2, CDA2, ARP2; HGNC:13203) has been reported to deaminate single-stranded (ss) and double-stranded (ds) DNA only^85^. AICDA-mediated RNA deamination has been reported in hepatitis B virus transcripts as RNA–DNA hybrids^19,86^. ApoB mRNA editing enzyme catalytic subunit 2 (APOBEC2, also known as ARCD1 and ARP1; HGNC:605) and apolipoprotein B mRNA editing enzyme catalytic polypeptide like 4 (APOBEC4, also known as MGC26594, FLJ25691 and RP1-127C7.4; HGNC:32152) have been reported to be inactive but retain their ability to bind to DNA^87^, suggesting that they may play a role in regulating DNA deamination by competing with their catalytically active paralogs. Of note, AICDA, APOBEC2 and APOBEC4 are also conserved in invertebrates, including sea urchin, nematodes and arthropods^88,89^.

Along with APOBEC1, ApoB mRNA editing enzyme catalytic subunit 3A (APOBEC3A, also known as ARP3 and PHRBN; HGNC:17343) and ApoB mRNA editing enzyme catalytic subunit 3G (APOBEC3G, also known as CEM15, MDS019, dJ494G10.1, FLJ12740, bK150C2.7; HGNC:17357) have been shown to deaminate both ssRNA and ss/dsDNA^15,19,73^. In placental mammals, there are a total of seven APOBEC3 paralogs, namely APOBEC3A–H^90^, and the five other APOBEC3 paralogs are thought to have lost the ability to conduct RNA deamination and editing^19^. Of note, APOBEC1 and APOBEC3 are conserved in vertebrates^88,91^, suggesting that C-to-U RNA editing may occur beyond placental mammals and also in jawless fish, zebrafish, *Xenopus* and other vertebrates.

APOBEC1, APOBEC3A and APOBEC3G each harbor similar yet distinct zinc-dependent cytidine deaminase domains (ZDDs), which underlie their specificity for different target dinucleotide sequences (see previous reviews for details^92^). The crystal structure of APOBEC1 revealed a dimer formation that extends the binding interface of RNA hairpin structures^93,94^. APOBEC1’s C-to-U RNA editing is regulated by cofactors such as A1CF^95^ (also known as ACF, ASP, ACF64, ACF65, APOBEC1CF; HGNC:24086), RBM47^96^ (also known as FLJ20273, NET18; HGNC:30358), RBM46^97^ (also known as MGC27016, CT68; HGNC:28401) and XPO1^93,94^ (also known as CRM1, CRM-1, emb; HGNC:12825). Both its NLS and NES domains are thought to control its nucleocytoplasmic cycle^98^. APOBEC3A targets and edits *SDHB* mRNAs and other transcripts in interferon-induced monocytes and macrophages^73^, but its crystal and NMR structures show that APOBEC3A favors ssDNA over RNA^99,100^, suggesting that RNA editing may not be the major enzymatic role of APOBEC3A. Interestingly, a few mutations in the active ZDD of APOBEC3A convert it into an RNA-specific C-to-U editor^101^. Unlike APOBEC1 and APOBEC3A, APOBEC3G has two distinct ZDD domains in tandem: the N-terminal ZDD domain is inactive but mainly responsible for DNA/RNA binding and oligomerization^102,103^, and the C-terminal ZDD domain is active and resembles the ZDD domain of APOBEC3A^99,100,104^. APOBEC3G also contains a cytoplasmic retention signal (CRS)^105^, suggesting that it may be the major C-to-U enzyme in cytoplasmic mRNA editing.

### Physiology and pathology of RNA deamination

RNA deamination expands the diversity of the transcriptome by post-transcriptionally editing both coding and noncoding RNAs^2,11,71^. The deaminated transcripts exhibit prominent differences in protein translation and molecular functions by the edited coding and noncoding sequences, respectively, which can profoundly influence cell and tissue physiology. Interestingly, RNA deaminations of some transcripts were shown to be essential and indispensable for maintaining normal and healthy physiological states, underscoring the necessity of understanding the physiological impacts of RNA deamination when investigating the relevant pathologies. In this section, how RNA deamination by ADARs and APOBECs contributes to different physiological contexts will be highlighted and outlined.

### Immunity

Among the ADAR family proteins, ADAR1 is known to play a crucial role in immunity through suppressing the aberrant immune responses. Mobile and repetitive genomic elements such as *Alu* sequences generate dsRNAs that activate dsRNA-sensing receptors—including melanoma differentiation-associated protein 5 (MDA5) and mitochondrial antiviral signaling protein (MAVS)^106^—which in turn trigger type I and III interferon signaling. ADAR1 edits the self-dsRNAs with Z conformation (Z-RNAs), which attenuates MDA5 binding to the self-dsRNAs by altering the secondary structure, eventually inhibiting antiviral and inflammatory responses^107^. A loss-of-function study on *ADAR1* supports this concept by demonstrating impaired editing of *Alu*-derived dsRNAs and consequent activation of MDA5^107,108^. Moreover, mutations in the Z-RNA binding and deaminase domains of *Adar1* have shown markedly elevated inflammation including interferon secretion^107,109,110^, underscoring the importance of the dsRNA recognition and deamination by the domains for immune regulation. Deletion of *Ifih1* (encoding MDA5) or *Mavs* rescues the dysregulated immune response observed in *Adar1* deficiency^111^, supporting the idea that ADAR1-mediated editing of Z-RNAs suppresses immune signaling, with downstream cascades mediated by these two dsRNA-binding proteins.

**Fig. 2:**
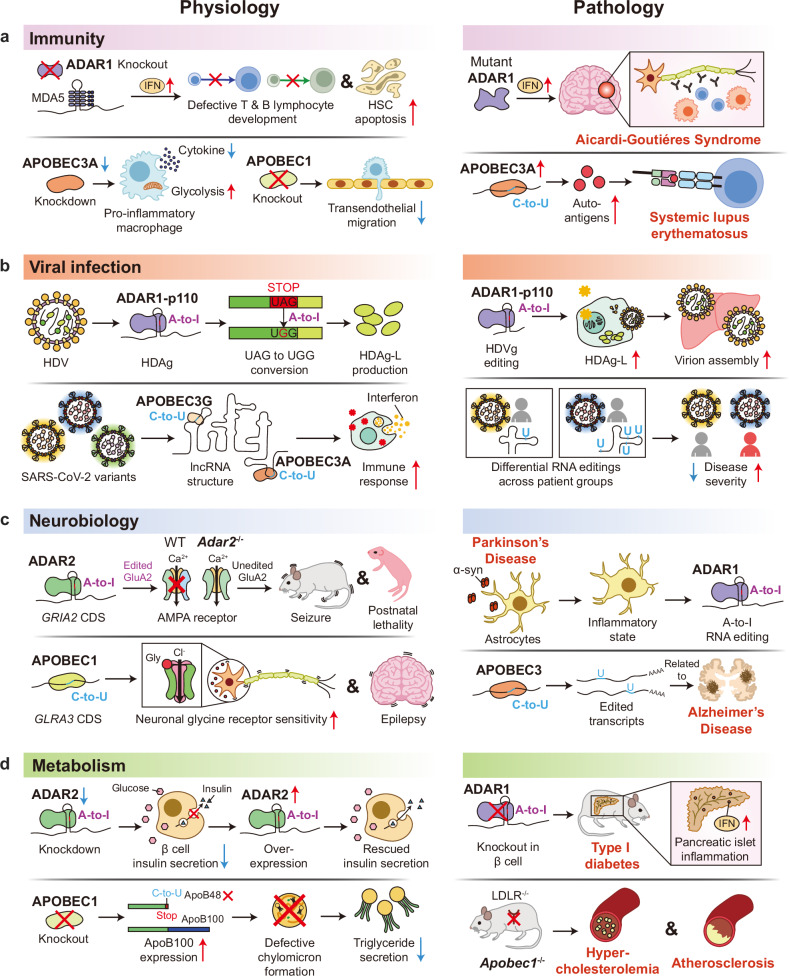
Physiological and pathological impacts of RNA editing. ADAR-mediated A-to-I RNA editing and APOBEC-mediated C-to-U RNA editing result in altered molecular functions of proteins and transcripts, substantially impacting the physiological processes. However, RNA editing can be dysregulated due to expressional change and mutations of editing enzymes. Consequently, aberrant RNA editing disrupts the physiological homeostasis and triggers the onset of pathology. **a** The development and essential immune functions of multiple immune cells are impaired, leading to disrupted immune homeostasis and the onset of autoimmunity. **b** Editing in virus RNA genome and host RNA induces recoded viral protein and altered host response, increasing the viral pathogenicity. **c** Misregulated editing in the transcripts encoding neurotransmitter receptors leads to neurological disorders, and evidence suggests the contribution of RNA editing in neurodegenerative diseases. **d** Metabolic processes such as insulin and triglyceride secretion are affected by RNA editing, contributing to metabolic disorders. Collectively, given the imperative role in physiology and contribution to the onset of pathology, RNA editing is a central biological process with potential implications in diseases and therapeutics.

Multiple studies have shown that A-to-I editing by ADAR1 is essential for maintaining immune homeostasis (Fig. 2a). Upon conditional knockout of *Adar1*, excessive interferon responses disrupt early and late development of T cells in thymus^5,112^ and late development of B cells in bone marrow^113^. Hematopoietic stem^114^ and progenitor cells^6^, as well as erythroid cells^115^, exhibited augmented innate immune signaling and apoptosis under *Adar1* deficiency. The abnormal development and survival of *Adar1*-deficient immune cells are primarily attributable to abolished editing of self-dsRNAs; however, one study reported hyper-edited adenosines in the 3′ UTR of erythroid-specific mRNAs^115^, suggesting additional molecular mechanisms through which A-to-I editing affects immune cell physiology.

Dysregulated interferon signaling caused by ADAR1 deficiency can lead to autoimmunity through chronic and systemic inflammation in both humans and mice^5^ (Fig. 2a). Aicardi–Goutières syndrome is a representative type I interferonopathy characterized by the impaired suppression of innate immune responses, which is attributed to dysfunctional ADAR1 harboring mutations in Z-nucleic acid binding and deaminase domains^116^. Mouse models with Aicardi–Goutières syndrome-associated mutations on Z-RNA binding (W197A^110^ and P195A^117^) or deaminase domains (D963H)^118^ recapitulated the autoimmune phenotypes, supporting the consistent pathology by the expression of the abnormal ADAR1 proteins.

In addition to A-to-I editing, C-to-U editing by APOBEC family proteins has also been observed in various immune cells (Fig. 2a). For instance, APOBEC3A catalyzes cytidine deamination of numerous transcripts in macrophages during M1 polarization and in monocytes upon hypoxia and interferon stimuli^73^. Another APOBEC family protein, APOBEC3G induces widespread RNA deamination in natural killer cells under hypoxic stress^119^. These observations allude to important roles of APOBEC-mediated deaminations in the physiology of the immune cells. In accordance with this idea, knockdown of *APOBEC3A* reduced cytokine secretion and increased glycolysis in pro-inflammatory macrophages^120^, and deletion of *Apobec1* increased phagocytosis and diminished migration of the innate immune cells^72^. Given that APOBEC-mediated RNA deamination is exquisitely controlled in the immune cells in response to external stimuli, excessive deamination has been suggested to deteriorate immune homeostasis. Elevated APOBEC3A-mediated RNA deamination has been observed in patients with systemic lupus erythematosus, implying a putative role of RNA deamination in autoantigen production^121^ (Fig. 2a). Further work is encouraged to explore the role of APOBEC-mediated RNA deamination in the development of autoimmunity and other immunological disorders.

### Viral infection

Given that dsRNAs targeted by ADAR1 are recognized by MDA5 and MAVS to activate antiviral signaling, A-to-I editing by ADAR1 has been thought to play a certain role in immune defense against viral infection. Indeed, ADAR1 deaminates viral RNAs by targeting their double-stranded features in several viruses, such as measles virus^122,123^, hepatitis delta virus^124^ and human immunodeficiency virus type 1 (HIV-1)^4^. However, ADAR1 can function as either an antiviral or a proviral factor depending on the context^4,124^.

In some cases, ADAR1 acts as a potent antiviral effector through extensive deamination. Hyperedited genomes of measles virus in subacute sclerosing panencephalitis brain tissues^122,123^ have been found to accumulate in infected tissues. These extensively edited RNAs may encode nonfunctional proteins or defective RNA species, limiting productive infection, although their precise contribution remains debated. Conversely, in other contexts, ADAR1 is co-opted by viruses to promote their life cycle through highly site-specific proviral editing. This complex interplay is further exemplified in hepatitis delta virus, where ADAR1 edits the amber/W site of the antigenome with high specificity^125^. The nuclear ADAR1-p110 converts a stop codon (UAG) into tryptophan (UGG) and enables the synthesis of large hepatitis delta antigen (HDAg-L), a protein required for virion assembly and packaging^124,125^ (Fig. 2b). This underscores that ADAR1 may facilitate viral propagation when its activity is precisely targeted.

Beyond direct editing of viral genomes, accumulating evidence indicates that ADAR1 dynamically reshapes host transcriptomes during viral infection. Transcriptome-wide analyses have shown that viral exposure reshapes A-to-I editing patterns in host RNAs, as observed in congenital cytomegalovirus and Zika virus infection^7^, consistent with ADAR1 involvement. Elevated or redistributed A-to-I editing signatures have also been described across diverse infections^4,126^. Such host-targeted A-to-I editing can affect splicing, recoding, RNA stability and the presentation of dsRNA structures to innate immune sensors^126^. In macrophages infected with HIV-1, for example, ADAR1-p150 edits host and viral dsRNAs in a manner that reduces their recognition by MDA5, thereby dampening interferon signaling^4,126^. These findings emphasize that ADAR1’s contribution to viral infection is not limited to modifying viral genomes but extends to dynamic remodeling of host RNA landscapes, further blurring the line between antiviral restriction and proviral adaptation.

In parallel, APOBEC family enzymes such as APOBEC3A, APOBEC3F and APOBEC3G are also strongly induced by interferons during viral infection. Their established role is the restriction of retroviruses such as HIV-1 through C-to-U deamination of nascent viral ssDNA^19^. However, this antiviral clarity becomes less certain in the context of RNA viruses. While APOBEC3 proteins are implicated in restricting hepatitis C virus^127^, whether this activity stems from direct RNA editing or editing-independent mechanisms has remained a topic of debate. A critical breakthrough in this area came from the coronavirus disease 2019 pandemic. Comparative analyses revealed a rampant C-to-U mutational signature in severe acute respiratory syndrome coronavirus 2 (SARS-CoV-2)^84^, accounting for approximately 55.8% of all mutations and representing a mutational footprint consistent with APOBEC-mediated viral RNA editing. Paradoxically, this same mechanism also acts as a primary source of viral genetic variation^19,84^, introducing sublethal mutations that can fuel viral adaptation and immune evasion^128^.

In addition to their direct effects on viral genomes, emerging evidence shows that APOBEC enzymes also induce widespread C-to-U RNA editing across the host transcriptome during viral infection^129^^,130^. This effect is observed in SARS-CoV-2 infection, where APOBEC3A and APOBEC3G contribute to differential C-to-U RNA editing in host long noncoding RNAs, which varies across viral variants^129^. Edited long noncoding RNAs interact with genes involved in immune and infection-related pathways. As such, C-to-U RNA editing of host RNAs may modulate host–pathogen interactions and the disease severity (Fig. 2b). However, the precise mechanisms governing this host-directed editing and its full functional consequences remain largely unclear. Nonetheless, this finding demonstrates that the role of APOBECs in viral infection is multifaceted, extending beyond direct viral restriction to encompass regulation of host immunity and generation of viral diversity. Elucidating the interplay between RNA editing systems and viral pathogens may offer potential avenues to design antiviral strategies or immune modulators.

### Neurobiology

ADAR2 is abundantly expressed in the brain and preferentially edits short dsRNA structures of specific transcripts, such as the Q/R site of *GRIA2* mRNA. Studies of Q/R site editing have established that ADAR2-mediated RNA deamination is indispensable for brain function (Fig. 2c). The Q/R site normally encodes a glutamine (Q) in the GluA2 subunit of AMPA-type glutamate receptors, which are essential for excitatory synaptic transmission. ADAR2-mediated editing converts this codon to arginine (R), and incorporation of the edited GluA2 subunit into AMPA receptors renders the channel impermeable to calcium^131^. Genetic disruption of the Q/R site in *Gria2* or deletion of *Adar2* resulted in severe neurological disorder and postnatal lethality^132,133^, underpinning the impact of the Q/R site deamination on neurological functions. Remarkably, expression of *Gria2* mutant encoding the edited version almost rescued the phenotypes of *Adar2*-knockout mice^133^, demonstrating that the single site is the most important substrate of ADAR2 for proper neurophysiology.

Beyond the Q/R site, evidence indicates that ADAR2 edits a variety of additional transcripts with potential physiological impact. Elevated ADAR2 expression and increased A-to-I editing of various transcripts have been reported in maturing neurons^8^, suggesting that ADAR2-mediated RNA editing contributes to neuronal development. Notably, the aforementioned mouse model (*Adar2*^*−/−*^*/Gria2*^*R/R*^), in which the impaired phenotypes under *Adar2* deficiency are rescued by a pre-edited *Gria2* allele, still exhibited multifaceted defects including abnormal behaviors and compromised sensations. These residual phenotypes point to the involvement of other edited transcripts to normal physiology^134^. More recently, ADAR2-dependent editing of multiple transcripts has been shown to fluctuate with circadian cycles, and *Adar2*-knockout mice display shortened circadian periods, implicating ADAR2-mediated editing in post-transcriptional regulation of circadian clockwork^135^. Although the precise contribution and mechanism of A-to-I editing of the individual transcripts remain incompletely understood, these studies collectively imply that ADAR2 is a multifaceted regulator of neuronal physiology, integrating RNA editing across diverse substrates to maintain brain homeostasis.

Although ADAR3 lacks catalytic activity, its exclusive expression in neurons suggests important roles in the nervous system. Consistently, mice lacking the *Adar3* exon exhibited dysregulation of genes associated with synaptic function in hippocampi, accompanied by impaired short-term and long-term memory formation upon contextual fear conditioning^136^, demonstrating its functional impact on the brain. Two models were proposed to explain the molecular action of ADAR3 contributing to neurophysiology. One model posits that ADAR3 can act as a competitive antagonist of catalytically active ADARs to inhibit A-to-I editing, which is supported by previous studies showing that ADAR3 blocks the substrates of ADAR1-^137^ and ADAR2-mediated deamination^138^. Another recent model suggests that ADAR3 exerts editing-independent functions, inhibiting translation and enhancing the stability of many mRNAs, although it remains unclear whether the functions are attributed to direct interactions between ADAR3 and target transcripts^139^.

Dysregulation of ADAR-mediated RNA editing has been proposed as a pathological feature of several neuroinflammatory diseases. In a Parkinson’s disease model, human induced pluripotent stem cell-derived neurons and astrocytes exposed to oligomeric alpha-synuclein revealed augmented and aberrant editing by ADAR1 in inflamed astrocytes^140^ (Fig. 2c). Similarly, in a sepsis-associated encephalopathy mouse model, acute neuroinflammation provokes substantial changes in A-to-I RNA editing in cerebral cells including microglia^141^, alluding to its potential role in the disease-associated pathophysiology. Although the precise mechanisms that link the altered RNA editing to the diseases remain elusive, these observations collectively suggest that tight regulation of ADAR-mediated RNA editing may be critical for preventing neurological dysfunction as well as maintaining normal neurophysiology. Not only A-to-I editing by ADARs but also C-to-U editing by APOBECs has been implicated in brain function and disease. The first evidence of APOBEC activity in the nervous system is the discovery of C-to-U editing in *GLRA3* mRNA, which substitutes a proline of glycine receptor alpha 3 subunit with a leucine (GlyRɑ3^P185L^), conferring increased glycine sensitivity to neurons^142^ (Fig. 2c). Subsequent studies demonstrated that the excessive *GLRA3* mRNA editing is closely associated with neuronal dysfunction and degeneration. Elevated APOBEC-mediated editing of *GLRA3* mRNA was observed in hippocampus of the patient with temporal lobe epilepsy, and ectopic expression of GlyRɑ3^P185L^ increased neuronal susceptibility to excitotoxicity^143^. Moreover, neuron type-specific expression of pre-edited mRNA encoding GlyRɑ3^P185L^ in mice disrupted cognitive function and fear memory formation^144^. In addition, a polymorphism in APOBEC1, which mediates C-to-U editing of *GLRA3* mRNA, has been proposed as a risk factor for epilepsy^145^. These findings exemplify the importance of finely tuned APOBEC activity in maintaining neuronal homeostasis. Genome/transcriptome-wide profiling and computational prediction of C-to-U editing by APOBECs revealed that differential editing of many transcripts is associated with neurological diseases, including Alzheimer’s disease^146^ as well as epilepsy^145,147^ (Fig. 2c). These studies suggest that dysregulated APOBEC-mediated editing of diverse transcripts may contribute to the onset or progression of neurological disorders. Consistent with this, mice with genetically inactivated *Apobec1* exhibited age-associated neurodegeneration featured by augmented neuroinflammation, aberrant myelination and lysosomal abnormalities in neurons and microglia, culminating in measurable declines in cognitive performance and motor activity^148^. Whether and how the individual transcripts that are edited by APOBEC family proteins in the nervous system participate in neurophysiology and neuropathology is yet to be appreciated, but it will be an intriguing arena to investigate in the future.

### Metabolism

Several studies demonstrated that RNA editing can act as a useful strategy to control metabolism in certain environments. Both ADAR2 expression and A-to-I editing are elevated in pancreatic islets of mice and pancreatic β cells upon high-fat diet and glucose stimulation^10^, implying a certain role of ADAR2-mediated editing responding to the metabolic conditions. In line with this observation, *Adar2* knockdown in pancreatic β cells and primary islets exhibited compromised insulin secretion that was restored by ectopic expression of ADAR2^149^, indicating that A-to-I editing by ADAR2 is actively involved in modulating metabolic signaling upon nutrient stimuli (Fig. 2d). Either insufficient or excessive A-to-I editing can contribute to the progression of metabolic diseases. About half of mice harboring *Adar1* deficiency in β cells developed type I diabetes, which is attributed to increased interferon signaling that is primarily triggered by the absence of A-to-I editing^150^ (Fig. 2d). Conversely, *Adar2* deficiency ameliorated glucose intolerance, insulin resistance and dyslipidemia in the liver of mice fed with high-fat diet^151^, suggesting that A-to-I editing by ADAR2 promotes the pathology of metabolic-associated fatty liver disease. Together, these findings underscore that the fine-tuned regulation of ADAR activity is not only essential for maintaining metabolic homeostasis but may also represent a potential therapeutic target in metabolic disorders.

C-to-U editing of apolipoprotein B (*APOB*) mRNA is one representative example of RNA editing shaping metabolism. The base substitution results in the production of a truncated form of APOB, ApoB48, from the edited transcript^15,18^. ApoB48 exerts an essential role in lipid metabolism by constituting chylomicrons^152^, triglyceride-storing particles for dietary fat transport. Studies using *Apobec1*-knockout mice underscore the impact of the APOBEC1-driven C-to-U editing by describing metabolic changes that are attributed to the abolished production of ApoB48^153^ (Fig. 2d). The mice exhibited abnormalities in lipoprotein metabolism, defects in chylomicron particle formation and triglyceride secretion^154,155^. Under metabolic stress such as low-density lipoprotein receptor deficiency and Western-type diet, the absence of editing markedly aggravated hypercholesterolemia and atherosclerosis^156^. These findings propose that *APOB* mRNA editing is critical for preventing metabolic diseases as well as maintaining lipid homeostasis. In addition to *Apob*, transcriptome-wide analyses in wild-type versus *Apobec1*-deficient mice have identified numerous putative target sites of *Apobec1*-mediated RNA editing^70^. However, the specific contributions of the individual transcripts that are presumably edited by APOBEC1 to metabolic pathways remain to be elucidated, representing an important avenue for future research.

### RNA deamination in cancer

**Fig. 3:**
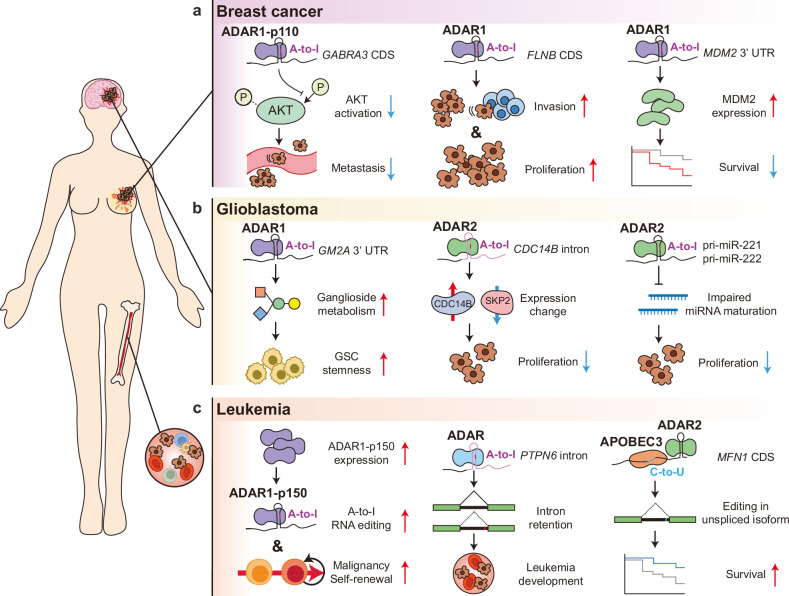
RNA editing shapes cancer phenotype through multiple mechanisms. Aberrant ADAR-mediated and APOBEC-mediated RNA editing modulates cancer phenotypes and influences clinical outcomes. Dysregulated RNA editing in coding sequences changes the cognate protein sequences, resulting in altered protein functions. Similarly, misregulated RNA editing in noncoding transcripts such as miRNAs impairs downstream gene regulation. As a result, aberrant RNA editing either exacerbates or suppresses the hallmarks of cancer, ultimately compromising or improving patient prognosis depending on the edited transcripts and cancer type. **a** In breast cancer, protein recoding by RNA editing alters the protein functions, influencing metastasis, invasion, proliferation and patient survival. **b** In GBM, RNA editing in UTRs or miRNAs modulates stemness of GSCs and proliferation of GBM cells. **c** In leukemia, RNA editing contributes to malignant reprogramming of myeloid progenitors, tumorigenesis and improved patient prognosis. Given its central role in cancer biology, RNA editing can serve as a promising prognostic marker for therapeutic strategy.

Both A-to-I and C-to-U RNA editing are considered to be dysregulated in cancer, leading to oncogenesis^157,158^, tumor progression^159,160^, immune evasion^161^, therapy resistance^162,163^ and other hallmarks of cancer^3^. This is achieved through the widespread expansion and alteration of RNA editing sites, largely driven by the misregulation of RNA editing enzymes and their cofactors^19,164^. Data analysis of 6,236 patient samples of 17 cancer types from The Cancer Genome Atlas (TCGA) led to the identification of thousands of A-to-I editing sites^14^. For example, 76,555 and 37,934 sites were discovered in breast invasive carcinoma and GBM, respectively^14^. Similar attempts have been conducted with C-to-U RNA editing but did not address existing computational issues in C-to-U site identification from sequencing data^71,74^. That being said, multiple genomic studies have reported APOBEC-related DNA mutation patterns in human cancer cell lines and cancers^21,165,166^, hinting at the possibility of concurrent RNA deamination in cancer via enhanced APOBEC activity. In fact, measuring C-to-U RNA editing activity has been shown to be effective for monitoring APOBEC3A activity in blood samples from patients with acute myeloid leukemia (AML) and myeloproliferative neoplasms^167^. The exact role of C-to-U editing in cancer biology requires further investigation by distinguishing the impact of DNA and RNA deamination and incorporating lessons from the successes and failures of studies in A-to-I RNA editing (Fig. 3).

### Breast cancer

One of the first studies on genome and transcriptome sequencing was on estrogen-receptor-positive (ER^+^) breast cancer (*n* = 1), which reported an elevated expression of *ADAR1* mRNA and 3,122 A-to-I editing sites in 1,637 transcripts^168^. Among these, 75 editing sites were validated by applying Sanger sequencing on both tumor DNA and RNA, including the nonsynonymous editing sites of *COG3* and *SRP9* transcripts. In this study, the mRNA levels of *ADAR2*, *ADAR3* and APOBEC RNA-editing enzymes (*APOBEC1*, *APOBEC3A* and *APOBEC3G*) were reported to be relatively low. However, large-scale RNA sequencing data analysis of breast invasive carcinoma samples (*n* = 1022) from TCGA and the International Cancer Genome Consortium showed moderate-to-high expression of *ADAR1*, *ADAR2* and *APOBEC3G* mRNAs^169^, indicating that both A-to-I and C-to-U RNA editing may play a role in other molecular subtypes of breast cancer.

Multiple large-scale studies have reported strong positive correlations between *ADAR1* mRNA levels and elevated A-to-I RNA editing in both coding and noncoding sites^14,170,171^ (Fig. 3a). In MCF7 and SKBR3 cells, *GABRA3* mRNA is edited via ADAR1-p110, and its recoded protein variant is thought to transdominantly suppress the function of nonrecoded GABRA3 proteins, resulting in the attenuation of breast cancer migration and metastasis^160^. ADAR1-mediated editing of *FLNB* mRNA promotes cell invasion and proliferation in BT20 and MDA-MB-468 cells^172^. ADAR1-mediated RNA editing of the 3′ UTR of *MDM2*, *GINS1* and *F11R* mRNAs and its subsequent enhanced protein expression was recently reported in MCF7 breast cancer cells^173^. It is yet to be determined whether some of these elevations in RNA editing are instead mediated by ADAR2 given that it is considered the major enzyme for mRNA recoding and not ADAR1. Regarding C-to-U RNA editing, overexpression of APOBEC3A in HEK293T cells led to the identification of recoding editing sites in breast-cancer-associated genes (*BARD1*, *PTEN* and *SF3B1*), which have been also validated by Sanger sequencing^146^. However, mRNA levels of APOBEC1 and APOBEC3A are relatively low in breast cancer, raising questions about whether APOBEC3A is the primary C-to-U RNA editing enzyme in this context. TCGA’s survival analysis indicates that *APOBEC3G* mRNA levels show favorable prognostic value in breast cancer, hinting at a potential link between APOBEC3G-mediated C-to-U RNA editing and breast cancer.

### Glioblastoma

GBM is the most common and aggressive form of malignant brain cancer with 6.8% of 5-year overall survival rate^174^, underscoring the urgent demand to elucidate the molecular mechanisms underlying its fatality. Multiple studies reported altered expression of ADARs and APOBECs in GBM and their association with the patient prognosis^175,176^, implicating dysregulated A-to-I and C-to-U RNA editing in GBM pathology. GBM stem cells (GSCs), a stem-like subpopulation in GBM, exhibit elevated ADAR1 expression and increased global A-to-I editing. Inhibiting ADAR1 activity impaired proliferation, self-renewal and tumorigenesis capacities of GSC, indicating the ADAR1 upregulation can support GBM growth. Ganglioside GM2 activator (*GM2A*) transcript was proposed as a target substrate responsible for ADAR1-dependent stem-like properties by enhancing ganglioside catabolism in GSCs^175^ (Fig. 3b). In contrast to ADAR1, ADAR2 is known for its antitumor effect on GBM. Both ADAR2 expression and A-to-I editing are reduced in GBM cells, and ADAR2-overexpressing GBM-derived U118 cells showed reduced proliferation in xenografted mice. It was shown that ADAR2 edits an intron of cell division cycle 14B (*CDC14B*) pre-mRNA, which increases CDC14B expression followed by cell cycle inhibition via reduced S-phase kinase associated protein 2 (SKP2)^177^ (Fig. 3b). In line with these findings, restoring *ADAR2* downregulation in multiple GBM cell lines attenuated cell proliferation and migration in its deaminase activity-dependent manner^178^. Other transcripts encoding neural proteins, such as GRIA2 and serotonin receptor 5HT_2C_ subunit, were underedited in GBM, suggesting that the compromised ADAR2-mediated RNA editing may contribute to GBM-associated neuronal dysfunction as well as tumor progression^179^. The tumor-suppressive effect of ADAR2 is not restricted to protein-coding mRNAs. ADAR2 edits the primary transcripts of several oncogenic miRNAs, such as miR-221 and miR-222, which inhibits maturation of the miRNAs, thereby reducing GBM cell proliferation^180^ (Fig. 3b).

Although GBM exhibited the second-highest C-to-U RNA editing (>2000 edited sites) among 22 cancer types^181^, the causal pathways by which the APOBEC-mediated RNA editing controls GBM development remain largely unexplored. The previous studies on C-to-U RNA editing by APOBECs in other tumors may propose plausible targets of APOBECs in GBM, such as neurofibromin 1 (*NF1*)^182^. C-to-U editing of *NF1* mRNA by APOBEC1 converts an arginine codon into a premature stop codon, yielding a truncated NF1 with compromised tumor-suppressor activity^183^. The pronounced editing of *NF1* was observed in cell lines originating from neurological cancers including GBM^182^. These observations suggest that the APOBEC-mediated *NF1* editing may influence the neuro-oncogenic malignancy. Further studies are required to define the roles of other individual transcripts edited by APOBECs in GBM, which can provide valuable insights into post-transcriptional regulation to control the pathogenesis and progression of GBM.

### Leukemia

Leukemia is a hematologic malignancy characterized by the overproduction of abnormal white blood cells in the bone marrow. These malignant cells deteriorate the immune homeostasis by diminishing the proportion of healthy immune cells. Given the aforementioned roles of RNA editing in immune cell physiology^5,120^, it is plausible that aberrant RNA editing can contribute to the pathogenesis and progression of leukemia^184,185^. Consistent with this, numerous studies demonstrated that exacerbated cancer phenotypes and poor clinical outcomes across the primary subtypes of leukemia are attributable to dysregulation of RNA editing^184,186^.

ADAR1 generally exhibits oncogenic activity in chronic leukemias. In chronic myeloid leukemia, elevated expression of ADAR1-p150 promotes malignant reprogramming of myeloid progenitors and self-renewal of leukemia stem cells^186^ (Fig. 3c). ADAR1 edits the primary transcripts of let-7 miRNAs, which hinders the maturation of the tumor-suppressive miRNA, enhancing the stem-like properties of chronic myeloid leukemia stem cells^185^. *ADAR1* knockout in the MEC1 cell line originating from chronic lymphocytic leukemia (CLL) cells reduces the proliferation and viability^184^, indicating that the oncogenic effect of ADAR1 is not restricted to leukemia of myeloid lineage. Integrative analysis of RNA sequencing and whole-exome sequencing data of samples from patients with CLL identified putative substrates of ADAR1 in the cancer. The editing of a subset of these transcripts is associated with CLL aggressiveness, which is defined by short time to first treatment^184^, alluding to the contribution of the edited transcripts to the malignancy.

Multifaceted involvements of ADAR- and APOBEC-mediated RNA editing in leukemias operate through diverse transcript-specific mechanisms. For example, in AML, A-to-I editing at an adenine (A^7866^) of mRNA encoding protein-tyrosine phosphatase non-receptor type 6 (*PTPN6*) is elevated, which leads to the accumulation of a splice variant with intron retention (Fig. 3c). Decrease of the aberrant isoform in the patients with AML at the remission stage supports the contribution of the specific post-transcriptional processing to leukemogenesis, although what ADAR family protein is responsible for the editing is unclear^187^.

In contrast to ADAR1, ADAR2 is recognized as a tumor-suppressive factor in certain subtypes of leukemia. ADAR2-mediated A-to-I conversions of component of oligomeric Golgi complex 3 (*COG3*) and coatomer subunit α (*COPA*) mRNAs are reported to suppress the growth of AML cell lines, Kasumi-1. In line with the finding, *ADAR2* is downregulated in patients with AML, and an AML mouse model transplanted with bone marrow cells overexpressing ADAR2 showed improved survival rates relative to mice transplanted with cells expressing a catalytically inactive mutant^188^, underpinning the anti-oncogenic effect of ADAR2-mediated editing.

APOBEC3 catalyzes extensive C-to-U RNA editing in hematopoietic cells including monocytes^73^ and is highly upregulated in AML, alluding to its functional relevance in leukemia biology. One recent study reported a recurrent C-to-U editing site on Mitofusin-1 (MFN1)-coding mRNA in CLL cohorts. Intriguingly, the C-to-U editing preferentially occurs in unspliced *MFN1* mRNA depending on ADAR2 as well as APOBEC3, implying cooperation between the two different deaminase family proteins on the intron-retained transcript^189^ (Fig. 3c). The C-to-U substitution converts serine into leucine at residue 329 of MFN1 protein, inhibiting the protein-mediated mitochondrial fusion^190^. The extent of *MFN1* C-to-U editing positively correlates with the clinical outcomes of patients with CLL^189^, suggesting its role in suppressing leukemia. Although this observation appears contradictory to the elevated expression of APOBEC3 in AML, it remains plausible, given that the specific C-to-U conversion requires ADAR2, a tumor suppressor in leukemia. Together, these findings demonstrate that ADAR- and APOBEC-mediated RNA editing promotes or restrains leukemia development via complex transcript-specific mechanisms.

## Concluding remarks

RNA editing has been implicated in a wide range of human diseases, including those affecting immunity, viral infection, neurobiology and metabolism, which we have discussed here from the perspectives of both A-to-I and C-to-U RNA editing. Other notable human diseases not covered in detail here, but nonetheless important, include enhanced editing of AZIN1 mRNA in hepatocellular carcinoma^158^, reduced editing of the 5-HT2C receptor in schizophrenia^191^ and correlation of editing activity and autism spectrum disorders^192^. This exploration of RNA editing in human diseases has been possible thanks to the early adaptation and incorporation of sequencing technology into clinical settings. Large-scale consortiums such as GTEx and TCGA have enabled the exploration of putative A-to-I and C-to-U editing sites and created or validated hypotheses in recoding and alternative noncoding regulation^11,29,14,181^. However, multiple data-analysis studies have raised concerns about the high potential for false-positive sites, highlighting the need for careful interpretation of sequencing data^74,193^. In fact, this concern in data analysis echoes similar issues raised in other RNA modifications such as m6A^194^, m1A^195^ and, more recently, ac4C^196^. In light of this, we recommend (1) site validation by Sanger sequencing of both DNA and RNA and (2) the development of computational tools with statistical guarantees. Myriads of putative A-to-I and C-to-U editing sites uncovered by the genome-wide searches raised two fundamental biological questions that should be resolved. First, how is editing at individual sites controlled? Although the identified editing loci varied across diverse biological and pathological contexts, the upstream mechanisms to determine whether and to what extent individual sites are edited remain largely elusive, with only a few exceptions. Which ADAR or APOBEC family protein is responsible for a substantial subset of editing sites remains unclear, as only a limited number of studies have directly verified enzyme dependencies using gain- or loss-of-function approaches. In addition, cofactors, such as DHX9^197^ and RBM47^96^, and yet-to-be-identified proteins may encourage or restrain the enzyme activities at specific sites. To understand the molecular landscape of site-specific A-to-I and C-to-U RNA editing, it is necessary to isolate and characterize ADAR- and APOBEC-containing complexes under the same conditions used in genome-wide surveys. Defining the RNA deaminase complexes implicated in the context-exclusive or preferential editing can lead to the delineation of entire molecular pathways that govern physiological or pathological controls of RNA editing.

The second unresolved question is whether and how the editing at individual loci influences physiology and pathology. Despite thousands of sites exhibiting differential editing by ADARs^14^ and APOBECs^181^ in certain contexts, the functional consequences of the individual edits are largely undefined. It has even been explicitly argued that the majority of them are functionally irrelevant^198^. Evolutionary analyses of human A-to-I^199^ and C-to-U editing sites^200^ revealed that they are generally not adaptive, suggesting that the edits hardly provide functional benefits. Despite these observations, some adaptive edits may have critical functions, given that they alter protein codes or RNA secondary structures. Abolishing the editing loci or expressing pre-edited transcripts can be an effective strategy to explore the molecular function of the individual site-specific editing, but it has been applied to fewer than 20 sites, underscoring the need for additional investigations. Such approaches will be valuable not only for identifying physiologically or pathologically relevant editing sites but also for elucidating the downstream events triggered by base substitutions at these loci. Together with resolving the first question, addressing this second issue will be essential to delineate the upstream and downstream molecular pathways of RNA editing in physiological and pathological contexts, which may provide potential therapeutic targets for human diseases that are associated with dysregulated RNA editing.
